# 3,3′-Di-*n*-butyl-1,1′-(*p*-phenyl­ene­dimethyl­ene)diimidazolium bis­(hexa­fluoro­phosphate)

**DOI:** 10.1107/S1600536810008536

**Published:** 2010-03-13

**Authors:** Rosenani A. Haque, Abbas Washeel, S. Fatimah Nasri, Chin Sing Yeap, Hoong-Kun Fun

**Affiliations:** a School of Chemical Science, Universiti Sains Malaysia, 11800 USM, Penang, Malaysia; b X-ray Crystallography Unit, School of Physics, Universiti Sains Malaysia, 11800 USM, Penang, Malaysia

## Abstract

The asymmetric unit of the title *N*-heterocyclic carbene compound, C_22_H_32_N_4_^2+^·2PF_6_^−^, consists of one half of the *N*-heterocyclic carbene dication and one hexa­fluoro­phosphate anion. The dication lies across a crystallographic inversion center. The imidazole ring is twisted away from the central benzene ring, making a dihedral angle of 76.23 (6)°. The hexa­fluoro­phosphate anions link the cations into a three-dimensional network *via* inter­molecular C—H⋯F hydrogen bonds. A weak C—H⋯π inter­action further stabilizes the crystal structure.

## Related literature

For background to *N*-heterocyclic carbenes, see: Arduengo *et al.* (1991 ▶); Papini *et al.* (2008 ▶). For applications of *N*-heterocyclic carbene derivatives, see: Meyer *et al.* (2009 ▶); Barnard *et al.* (2004 ▶); Lin & Vasam (2007 ▶). For a related structure, see: Washeel *et al.* (2010 ▶). For the stability of the temperature controller used for the data collection, see: Cosier & Glazer (1986 ▶).
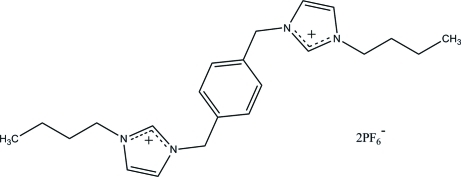


## Experimental

### 

#### Crystal data

C_22_H_32_N_4_^2+^·2PF_6_^−^
*M* *_r_* = 642.46
Monoclinic, 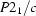

*a* = 8.9802 (5) Å
*b* = 17.8421 (10) Å
*c* = 9.3637 (5) Å
β = 113.233 (1)°
*V* = 1378.64 (13) Å^3^
*Z* = 2
Mo *K*α radiation
μ = 0.26 mm^−1^
*T* = 100 K
0.37 × 0.25 × 0.20 mm

#### Data collection

Bruker APEX Duo CCD area detector diffractometer
Absorption correction: multi-scan (*SADABS*; Bruker, 2009 ▶) *T*_min_ = 0.910, *T*_max_ = 0.950
21938 measured reflections
5550 independent reflections
4750 reflections with *I* > 2σ(*I*)
*R*_int_ = 0.027

#### Refinement

*R*[*F* ^2^ > 2σ(*F* ^2^)] = 0.036
*wR*(*F* ^2^) = 0.121
*S* = 1.10
5550 reflections
190 parameters
H atoms treated by a mixture of independent and constrained refinement
Δρ_max_ = 0.52 e Å^−3^
Δρ_min_ = −0.35 e Å^−3^

### 

Data collection: *APEX2* (Bruker, 2009 ▶); cell refinement: *SAINT* (Bruker, 2009 ▶); data reduction: *SAINT*; program(s) used to solve structure: *SHELXTL* (Sheldrick, 2008 ▶); program(s) used to refine structure: *SHELXTL*; molecular graphics: *SHELXTL*; software used to prepare material for publication: *SHELXTL* and *PLATON* (Spek, 2009 ▶).

## Supplementary Material

Crystal structure: contains datablocks global, I. DOI: 10.1107/S1600536810008536/sj2739sup1.cif

Structure factors: contains datablocks I. DOI: 10.1107/S1600536810008536/sj2739Isup2.hkl

Additional supplementary materials:  crystallographic information; 3D view; checkCIF report

## Figures and Tables

**Table 1:** Hydrogen-bond geometry (Å, °) Table 1 ▶. Hydrogen bond geometry (Å, °). *Cg*1 is the centroid of the C1–C3,C1*A*–C3*A* benzene ring.

*D*—H⋯*A*	*D*—H	H⋯*A*	*D*⋯*A*	*D*—H⋯*A*
C1—H1*A*⋯F3^i^	1.004 (17)	2.532 (18)	3.3945 (14)	143.8 (15)
C4—H4*A*⋯F4^i^	0.97	2.52	3.3516 (14)	144
C4—H4*B*⋯F2^ii^	0.97	2.45	3.3497 (14)	153
C7—H7*A*⋯F1^iii^	0.93	2.36	2.8798 (13)	115
C8—H8*B*⋯F6^iv^	0.97	2.49	3.3537 (13)	148
C8—H8*A*⋯*Cg*1^v^	0.97	2.84	3.7376 (12)	154
C8—H8*A*⋯*Cg*1^vi^	0.97	2.84	3.7376 (12)	154

Symmetry codes: (i) 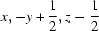; (ii) 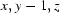; (iii) 
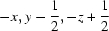
; (iv) 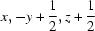; (v) 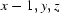; (vi) 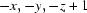.

**Fig. 1.**
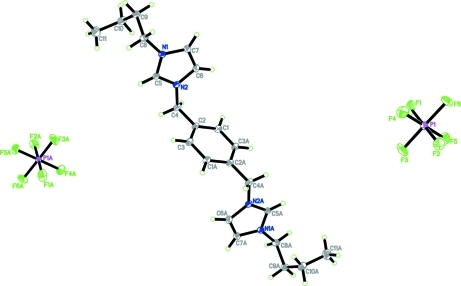
The molecular structure of the title compound with 50% probability ellipsoids for non-H atoms. Atoms with suffix A are generated by the symmetry operation (1-x, -y, 1-z).

**Fig. 2.**
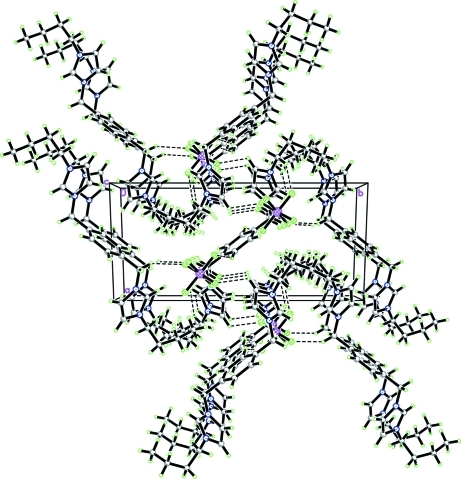
Crystal packing of the title compound, viewed down the c axis, showing the molecules linked into a 3-D network. Intermolecular hydrogen bonds are shown as dashed lines.

## supplementary crystallographic information

### Comment

*N*-heterocyclic carbene (NHC) ligands have enjoyed wide applicability as ligands for transition and main group metals since the first crystalline free carbene were isolated in 1991 by Arduengo and co-workers (Arduengo *et al.*, 1991). They display rich coordination chemistry and are able to form stable complexes with a large number of transition metals in both high and low oxidation states (Papini *et al.*, 2008). The complexes are widely used in catalysis and are useful in medicinal science applications (Meyer *et al.*, 2009; Barnard *et al.*, 2004; Lin & Vasam, 2007). These compounds show unusually high thermal stability and nucleophilic behavior, in part due to the analogy of *N*-heterocyclic carbenes with strong Lewis-basic phosphines. NHCs are also cheap, non-toxic and easily prepared as an azolium salt precursor (Papini *et al.*, 2008).

The asymmetric unit of the title compound consists of half of the *N*-heterocyclic carbene dication and one hexafluorophosphate anion (Fig. 1). The dication lies across a crystallograpic inversion center. The geometrical parameters are comparable to its related structure (Washeel *et al.*, 2010). The imidazole ring (N1–C5–N2–C7–C6) is planar with a maximum deviation of 0.003 (1) Å for atom C6 and is twisted away from the central benzene ring making a dihedral angle of 76.23 (6)°. The hexafluorophosphate anions linked the molecules into a three-dimensional network *via* intermolecular C—H···F hydrogen bonds (Fig. 2, Table 1). Short intermolecular F1···C5 and F1···C7 of 2.8636 (12) and 2.8798 (13) Å contacts are observed. A weak C–H···π interaction further stabilizes the crystal structure (Table 1).

### Experimental

To a solution of *p*-xylylene dichloride (1 g, 5.75 mmol) in 30 ml of 1,4-dioxane, 1-butylimidazole (1.42 g, 11.5 mmol) was added. The mixture was refluxed at 373 K for 24 h. The slurry product was isolated by decantation then washed with diethyl ether (2x3 ml). KPF_6_ (2.1 g, 11.5 mmol) in 20 ml of distilled water was then added with stirring for 1 h and the suspension was left standing overnight. The white precipitate was filtered, washed with distilled water several times and recrystallized from acetonitrile. The yield was found to be 2.30 g (62.7 %), m.p.: 411-413 K. Crystals suitable for X-ray was obtained by slow evaporation of the salt solution in acetonitrile at 281 K.

### Refinement

The H1A and H3A hydrogen atoms were located from a difference Fourier map and refined freely. The remaining H atoms were positioned geometrically and refined using a riding model, with C–H = 0.93 or 0.97 Å and *U*_iso_(H) = 1.2 or 1.5 *U*_eq_(C). A rotating group model was applied for the methyl groups.  Short intermolecular F1···C5 and F1···C7 of 2.8636 (12) and 2.8798 (13) Å contacts are observed.

### Crystal data

**Table:**

C_22_H_32_N_4_^2+^·2PF_6_^−^	*F*(000) = 660
*M**_r_* = 642.46	*D*_x_ = 1.548 Mg m^−^^3^
Monoclinic, *P*2_1_/*c*	Mo *K*α radiation, λ = 0.71073 Å
Hall symbol: -P 2ybc	Cell parameters from 8699 reflections
*a* = 8.9802 (5) Å	θ = 3.3–35.1°
*b* = 17.8421 (10) Å	µ = 0.26 mm^−^^1^
*c* = 9.3637 (5) Å	*T* = 100 K
β = 113.233 (1)°	Block, colourless
*V* = 1378.64 (13) Å^3^	0.37 × 0.25 × 0.20 mm
*Z* = 2	

### Data collection

**Table:**

Bruker APEX Duo CCD area detector diffractometer	5550 independent reflections
Radiation source: fine-focus sealed tube	4750 reflections with *I* > 2σ(*I*)
graphite	*R*_int_ = 0.027
φ and ω scans	θ_max_ = 34.0°, θ_min_ = 3.3°
Absorption correction: multi-scan (*SADABS*; Bruker, 2009)	*h* = −12→14
*T*_min_ = 0.910, *T*_max_ = 0.950	*k* = −25→28
21938 measured reflections	*l* = −14→14

### Refinement

**Table:**

Refinement on *F*^2^	Primary atom site location: structure-invariant direct methods
Least-squares matrix: full	Secondary atom site location: difference Fourier map
*R*[*F*^2^ > 2σ(*F*^2^)] = 0.036	Hydrogen site location: inferred from neighbouring sites
*wR*(*F*^2^) = 0.121	H atoms treated by a mixture of independent and constrained refinement
*S* = 1.10	*w* = 1/[σ^2^(*F*_o_^2^) + (0.0669*P*)^2^ + 0.3404*P*] where *P* = (*F*_o_^2^ + 2*F*_c_^2^)/3
5550 reflections	(Δ/σ)_max_ < 0.001
190 parameters	Δρ_max_ = 0.52 e Å^−^^3^
0 restraints	Δρ_min_ = −0.35 e Å^−^^3^

### Special details

**Table:**

Experimental. The crystal was placed in the cold stream of an Oxford Cryosystems Cobra open-flow nitrogen cryostat (Cosier & Glazer, 1986) operating at 100.0 (1) K.
Geometry. All esds (except the esd in the dihedral angle between two l.s. planes) are estimated using the full covariance matrix. The cell esds are taken into account individually in the estimation of esds in distances, angles and torsion angles; correlations between esds in cell parameters are only used when they are defined by crystal symmetry. An approximate (isotropic) treatment of cell esds is used for estimating esds involving l.s. planes.
Refinement. Refinement of *F*^2^ against ALL reflections. The weighted *R*-factor wR and goodness of fit *S* are based on *F*^2^, conventional *R*-factors *R* are based on *F*, with *F* set to zero for negative *F*^2^. The threshold expression of *F*^2^ > σ(*F*^2^) is used only for calculating *R*-factors(gt) etc. and is not relevant to the choice of reflections for refinement. *R*-factors based on *F*^2^ are statistically about twice as large as those based on *F*, and *R*- factors based on ALL data will be even larger.

### Fractional atomic coordinates and isotropic or equivalent isotropic displacement parameters (Å^2^)

**Table:**

	*x*	*y*	*z*	*U*_iso_*/*U*_eq_	
P1	0.24993 (3)	0.644667 (14)	0.41525 (3)	0.01504 (7)	
F1	0.17538 (13)	0.58756 (4)	0.27368 (9)	0.0377 (2)	
F2	0.32152 (11)	0.70282 (5)	0.55641 (8)	0.03461 (19)	
F3	0.40070 (10)	0.59059 (5)	0.49878 (11)	0.0363 (2)	
F4	0.15300 (10)	0.60287 (5)	0.50374 (10)	0.03387 (18)	
F5	0.34534 (10)	0.68616 (5)	0.32536 (10)	0.03212 (18)	
F6	0.09980 (9)	0.70032 (5)	0.33174 (9)	0.02912 (16)	
N1	−0.09308 (9)	−0.12292 (5)	0.43634 (9)	0.01428 (14)	
N2	0.12122 (9)	−0.11365 (5)	0.38494 (10)	0.01434 (14)	
C1	0.42820 (12)	−0.01546 (6)	0.34135 (11)	0.01608 (16)	
C2	0.39498 (11)	−0.06136 (5)	0.44620 (11)	0.01435 (15)	
C3	0.46724 (12)	−0.04565 (6)	0.60501 (11)	0.01585 (16)	
C4	0.28430 (11)	−0.12854 (6)	0.38858 (12)	0.01716 (17)	
H4A	0.2753	−0.1415	0.2849	0.021*	
H4B	0.3314	−0.1710	0.4560	0.021*	
C5	0.05423 (11)	−0.15095 (5)	0.46797 (11)	0.01380 (15)	
H5A	0.1022	−0.1900	0.5365	0.017*	
C6	0.01313 (13)	−0.05969 (6)	0.29775 (13)	0.01997 (18)	
H6A	0.0295	−0.0256	0.2301	0.024*	
C7	−0.12157 (12)	−0.06587 (6)	0.32955 (13)	0.01986 (18)	
H7A	−0.2152	−0.0371	0.2873	0.024*	
C8	−0.20778 (12)	−0.14960 (6)	0.50215 (12)	0.01732 (17)	
H8A	−0.2538	−0.1069	0.5344	0.021*	
H8B	−0.1501	−0.1799	0.5934	0.021*	
C9	−0.34402 (11)	−0.19587 (6)	0.38501 (12)	0.01788 (17)	
H9A	−0.4209	−0.2086	0.4304	0.021*	
H9B	−0.4004	−0.1652	0.2940	0.021*	
C10	−0.28848 (13)	−0.26785 (6)	0.33359 (13)	0.02143 (19)	
H10A	−0.3792	−0.2892	0.2475	0.026*	
H10B	−0.2049	−0.2557	0.2961	0.026*	
C11	−0.22253 (16)	−0.32643 (7)	0.46144 (18)	0.0314 (3)	
H11B	−0.1973	−0.3714	0.4194	0.047*	
H11C	−0.3025	−0.3372	0.5028	0.047*	
H11D	−0.1262	−0.3076	0.5428	0.047*	
H1A	0.372 (2)	−0.0256 (10)	0.227 (2)	0.026 (4)*	
H3A	0.447 (2)	−0.0786 (10)	0.671 (2)	0.025 (4)*	

### Atomic displacement parameters (Å^2^)

**Table:**

	*U*^11^	*U*^22^	*U*^33^	*U*^12^	*U*^13^	*U*^23^
P1	0.01633 (12)	0.01343 (12)	0.01512 (11)	0.00062 (7)	0.00596 (9)	0.00052 (7)
F1	0.0666 (6)	0.0170 (3)	0.0251 (4)	−0.0065 (3)	0.0134 (4)	−0.0070 (3)
F2	0.0451 (5)	0.0276 (4)	0.0184 (3)	−0.0044 (3)	−0.0011 (3)	−0.0044 (3)
F3	0.0324 (4)	0.0346 (4)	0.0439 (5)	0.0182 (3)	0.0173 (3)	0.0191 (4)
F4	0.0300 (4)	0.0430 (5)	0.0339 (4)	−0.0048 (3)	0.0182 (3)	0.0085 (3)
F5	0.0322 (4)	0.0337 (4)	0.0369 (4)	−0.0006 (3)	0.0204 (3)	0.0123 (3)
F6	0.0231 (3)	0.0298 (4)	0.0278 (3)	0.0112 (3)	0.0029 (3)	0.0008 (3)
N1	0.0115 (3)	0.0145 (3)	0.0169 (3)	0.0003 (2)	0.0056 (3)	0.0004 (3)
N2	0.0127 (3)	0.0128 (3)	0.0185 (3)	−0.0015 (2)	0.0071 (3)	−0.0015 (3)
C1	0.0163 (4)	0.0168 (4)	0.0167 (4)	−0.0029 (3)	0.0081 (3)	−0.0029 (3)
C2	0.0125 (3)	0.0133 (4)	0.0192 (4)	−0.0020 (3)	0.0083 (3)	−0.0030 (3)
C3	0.0168 (4)	0.0158 (4)	0.0178 (4)	−0.0029 (3)	0.0099 (3)	−0.0010 (3)
C4	0.0147 (4)	0.0144 (4)	0.0258 (4)	−0.0035 (3)	0.0116 (3)	−0.0058 (3)
C5	0.0118 (3)	0.0133 (4)	0.0164 (4)	−0.0002 (3)	0.0056 (3)	−0.0003 (3)
C6	0.0180 (4)	0.0168 (4)	0.0248 (4)	−0.0006 (3)	0.0082 (3)	0.0057 (3)
C7	0.0156 (4)	0.0163 (4)	0.0265 (5)	0.0023 (3)	0.0070 (3)	0.0057 (3)
C8	0.0142 (4)	0.0214 (4)	0.0186 (4)	−0.0014 (3)	0.0089 (3)	−0.0021 (3)
C9	0.0124 (4)	0.0197 (4)	0.0218 (4)	−0.0007 (3)	0.0071 (3)	−0.0008 (3)
C10	0.0196 (4)	0.0212 (5)	0.0263 (5)	−0.0028 (3)	0.0121 (4)	−0.0044 (4)
C11	0.0290 (6)	0.0223 (5)	0.0447 (7)	0.0055 (4)	0.0166 (5)	0.0043 (5)

### Geometric parameters (Å, °)

**Table:**

P1—F3	1.5939 (8)	C4—H4A	0.9700
P1—F1	1.5948 (8)	C4—H4B	0.9700
P1—F5	1.5999 (8)	C5—H5A	0.9300
P1—F2	1.6020 (8)	C6—C7	1.3595 (15)
P1—F4	1.6029 (8)	C6—H6A	0.9300
P1—F6	1.6073 (7)	C7—H7A	0.9300
N1—C5	1.3337 (12)	C8—C9	1.5241 (14)
N1—C7	1.3782 (13)	C8—H8A	0.9700
N1—C8	1.4725 (12)	C8—H8B	0.9700
N2—C5	1.3335 (12)	C9—C10	1.5229 (15)
N2—C6	1.3822 (13)	C9—H9A	0.9700
N2—C4	1.4755 (12)	C9—H9B	0.9700
C1—C3^i^	1.3966 (13)	C10—C11	1.5222 (17)
C1—C2	1.3981 (13)	C10—H10A	0.9700
C1—H1A	1.005 (17)	C10—H10B	0.9700
C2—C3	1.3964 (13)	C11—H11B	0.9600
C2—C4	1.5141 (13)	C11—H11C	0.9600
C3—C1^i^	1.3966 (13)	C11—H11D	0.9600
C3—H3A	0.925 (17)		
			
F3—P1—F1	91.03 (5)	H4A—C4—H4B	107.9
F3—P1—F5	90.61 (5)	N2—C5—N1	108.63 (8)
F1—P1—F5	89.70 (5)	N2—C5—H5A	125.7
F3—P1—F2	90.02 (5)	N1—C5—H5A	125.7
F1—P1—F2	178.91 (5)	C7—C6—N2	107.00 (9)
F5—P1—F2	90.60 (5)	C7—C6—H6A	126.5
F3—P1—F4	89.66 (5)	N2—C6—H6A	126.5
F1—P1—F4	89.82 (5)	C6—C7—N1	106.99 (9)
F5—P1—F4	179.45 (5)	C6—C7—H7A	126.5
F2—P1—F4	89.87 (5)	N1—C7—H7A	126.5
F3—P1—F6	179.09 (5)	N1—C8—C9	111.68 (8)
F1—P1—F6	89.64 (5)	N1—C8—H8A	109.3
F5—P1—F6	88.78 (4)	C9—C8—H8A	109.3
F2—P1—F6	89.31 (4)	N1—C8—H8B	109.3
F4—P1—F6	90.95 (5)	C9—C8—H8B	109.3
C5—N1—C7	108.79 (8)	H8A—C8—H8B	107.9
C5—N1—C8	125.60 (8)	C10—C9—C8	114.48 (8)
C7—N1—C8	125.60 (8)	C10—C9—H9A	108.6
C5—N2—C6	108.59 (8)	C8—C9—H9A	108.6
C5—N2—C4	124.66 (8)	C10—C9—H9B	108.6
C6—N2—C4	126.75 (8)	C8—C9—H9B	108.6
C3^i^—C1—C2	120.28 (9)	H9A—C9—H9B	107.6
C3^i^—C1—H1A	120.5 (10)	C11—C10—C9	113.87 (9)
C2—C1—H1A	119.2 (10)	C11—C10—H10A	108.8
C3—C2—C1	119.47 (8)	C9—C10—H10A	108.8
C3—C2—C4	120.16 (9)	C11—C10—H10B	108.8
C1—C2—C4	120.36 (8)	C9—C10—H10B	108.8
C2—C3—C1^i^	120.25 (9)	H10A—C10—H10B	107.7
C2—C3—H3A	117.1 (11)	C10—C11—H11B	109.5
C1^i^—C3—H3A	122.5 (11)	C10—C11—H11C	109.5
N2—C4—C2	111.82 (8)	H11B—C11—H11C	109.5
N2—C4—H4A	109.3	C10—C11—H11D	109.5
C2—C4—H4A	109.3	H11B—C11—H11D	109.5
N2—C4—H4B	109.3	H11C—C11—H11D	109.5
C2—C4—H4B	109.3		
			
C3^i^—C1—C2—C3	−0.02 (16)	C8—N1—C5—N2	178.85 (8)
C3^i^—C1—C2—C4	−178.77 (9)	C5—N2—C6—C7	−0.48 (12)
C1—C2—C3—C1^i^	0.02 (16)	C4—N2—C6—C7	179.81 (9)
C4—C2—C3—C1^i^	178.77 (9)	N2—C6—C7—N1	0.50 (12)
C5—N2—C4—C2	−118.10 (10)	C5—N1—C7—C6	−0.35 (12)
C6—N2—C4—C2	61.57 (13)	C8—N1—C7—C6	−179.15 (9)
C3—C2—C4—N2	78.63 (11)	C5—N1—C8—C9	−104.24 (11)
C1—C2—C4—N2	−102.63 (10)	C7—N1—C8—C9	74.36 (12)
C6—N2—C5—N1	0.26 (11)	N1—C8—C9—C10	63.05 (11)
C4—N2—C5—N1	179.98 (8)	C8—C9—C10—C11	67.80 (12)
C7—N1—C5—N2	0.05 (11)		

Symmetry codes: (i) −*x*+1, −*y*, −*z*+1.

### Hydrogen-bond geometry (Å, °)

**Table:**

Table 1. Hydrogen bond geometry (Å, °). Cg1 is centroids of benzene ring C1-C2-C3-C1A-C2A-C3A.

**Table:**

*D*—H···*A*	*D*—H	H···*A*	*D*···*A*	*D*—H···*A*
C1—H1A···F3^ii^	1.004 (17)	2.532 (18)	3.3945 (14)	143.8 (15)
C4—H4A···F4^ii^	0.97	2.52	3.3516 (14)	144
C4—H4B···F2^iii^	0.97	2.45	3.3497 (14)	153
C7—H7A···F1^iv^	0.93	2.36	2.8798 (13)	115
C8—H8B···F6^v^	0.97	2.49	3.3537 (13)	148
C8—H8A···Cg1^vi^	0.97	2.84	3.7376 (12)	154
C8—H8A···Cg1^vii^	0.97	2.84	3.7376 (12)	154

Symmetry codes: (ii) *x*, −*y*+1/2, *z*−1/2; (iii) *x*, *y*−1, *z*; (iv) −*x*, *y*−1/2, −*z*+1/2; (v) *x*, −*y*+1/2, *z*+1/2; (vi) *x*−1, *y*, *z*; (vii) −*x*, −*y*, −*z*+1.
